# Management of oroantral communication using buccal advanced flap

**DOI:** 10.11604/pamj.2019.34.69.19959

**Published:** 2019-10-03

**Authors:** Akram Belmehdi, Karima El Harti

**Affiliations:** 1 Oral surgeon, Dental Center of Treatment and Diagnosis Ibn Sina Hospital, Rabat, Morocco; 2 Oral Surgery, Faculty of Dentistry of Rabat, Mohammed V University, Morocco

**Keywords:** Oroantral fistula, oroantral communication, buccal advancement flap, maxillary sinus

## Abstract

Oroantral communication (OAC) or fistula (OAF) is an open pathological communication between the oral cavity and maxillary sinus which mostly occurs as a result of extraction of upper molars and premolars, iatrogenic complications or from dental infections, osteomyelitis, radiation therapy or trauma. Several alternative techniques modalities have been described throughout the years for the management of OAC and OAF which show both advantages and limitations. The most employed surgical flaps are of three types: advanced buccal flap, palatal flap and buccal fat pad flap. The authors present two clinical cases: oroantral communication and oroantral fistula, both were treated by using buccal advancement flap.

## Introduction

Oroantral communication (OAC) is the space created between the maxillary sinus and the oral cavity, which, if not treated, will progress to oroantral fistula (OAF) or chronic sinus disease [1]. These complications occur most commonly during extraction of upper molar and premolar teeth (48%). The major reason is the anatomic proximity or projection of the roots within the maxillary sinus [2, 3]. Other causes of OAC/OAF include tuberosity fracture, dentoalveolar/periapical infections of molars, implant dislodgement into maxillary sinus, trauma (7.5%), presence of maxillary cysts or tumors (18.5%), osteoradionecrosis, flap necrosis, dehiscence following implant failure and sometimes as a complication of the Caldwell-Luc procedure [2-4]. OACs may close spontaneously especially when the defect has a size smaller than 5mm. Nevertheless to our knowledge, it has never been actually proven that small OACs (-5mm) will heal by themselves. Also, it is difficult to determine the size of the OAC clinically. To prevent chronic sinusitis and the development of fistulas, it is generally accepted that all of these defects should be closed within 24 to 48 hours [5]. Decision on how to treat an OAC should be based on the size of communication, time of diagnosis, and presence of an infection. Furthermore, the selection of treatment strategy is influenced by the amount and condition of tissue available for repair and the possible placement of dental implants in the future [1, 6]. Many techniques to close OAC/OAF have been described in the literature, such as buccal flap, palatal flap, buccal fat pad and relate modifications. They are their own advantages and disadvantages depending on the cases and the size of the defects occurred. Most of them rely on mobilizing the tissue and advancing the resultants flap into defect [1-5]. The goal of this paper is to reports two cases of OAC/OAF which have been successfully treated by surgical buccal advancement flap technique.

## Patient and observation

**Figure 1:**
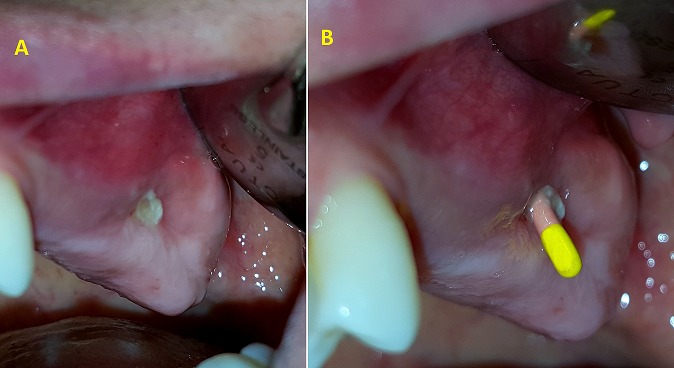
A) intraoperative picture showing oroantral fistula; B) use of the gutta-percha cone to explore the fistula by X-ray

**Figure 2:**
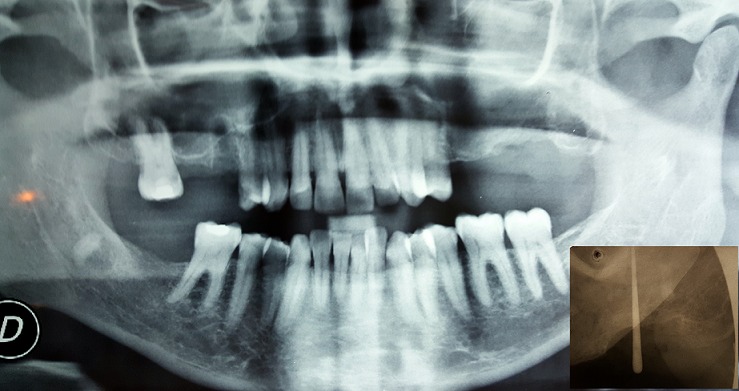
Panoramic x-ray showing an osseous defect in the left upper molar region. The Fistulography bellow confirmed the oroantral communication

**Figure 3:**
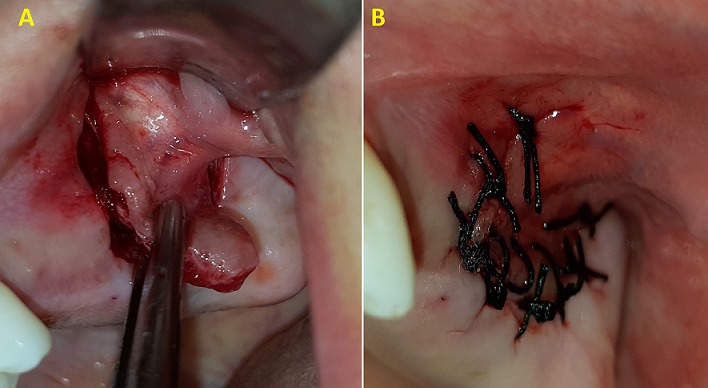
A) intraoperative view of the surgical procedure of the vestibular advanced flap; B) the buccal flap was placed over the defect and sutured

**Figure 4:**
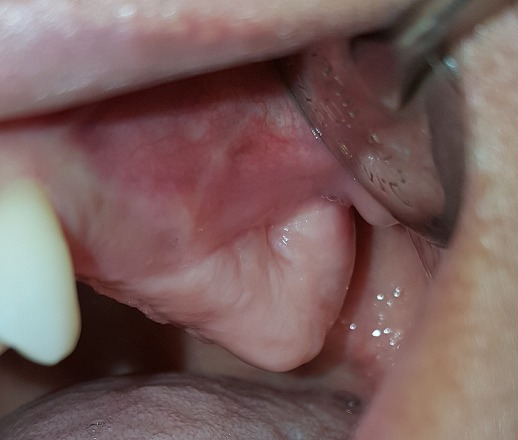
Postoperative healing after 6 months

**Case 1:** a healthy 45 years old female complaining of discomfort on the left hemi face and of persistence of non-healed oral fistula due to a maxillary second molar extraction one year before. Clinical (Figure 1) and radiographic investigation (Figure 2) revealed oroantral communication in the vestibular ridge. There was no discharge from the fistula or any signs of acute infection. Treatment plan has been explained to the patient, and pre-operative medications were advised. Surgery was done on the next week under local anesthesia; two vertical releasing incisions having a trapezoidal shape were placed, and buccal flap was raised (Figure 3 A). The buccal flap was placed over the defect and sutured (Figure 3 B), and routine postoperative instructions with prescription of antibiotics and analgesics were given to the patient. The patient was scheduled for regular follow up appointments, and healing was uneventful after six months (Figure 4).

**Figure 5:**
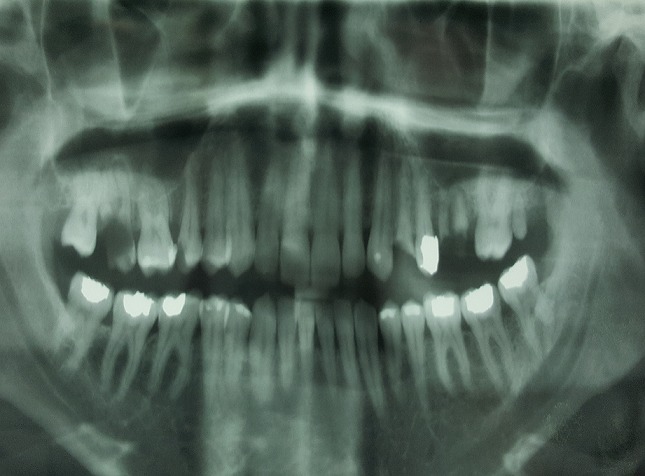
Orthopantomogram of the patient

**Figure 6:**
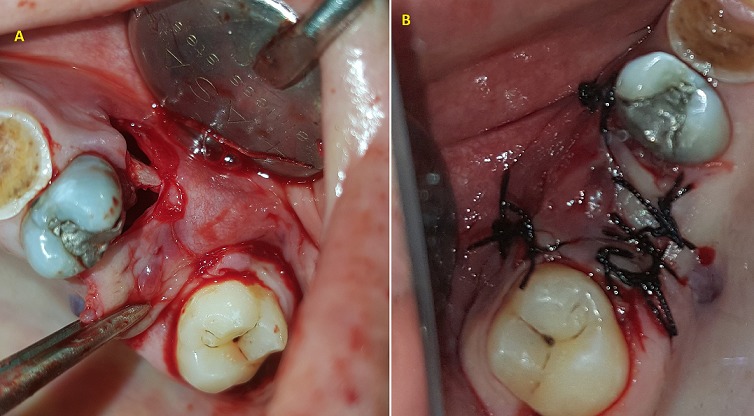
A) preparation of the buccal advanced flap; B) suture of the flap

**Figure 7:**
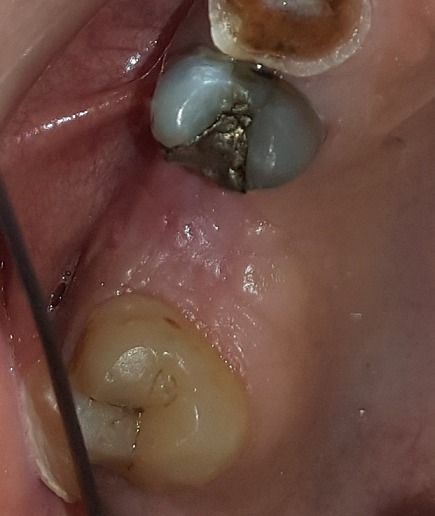
Follow-up after six months

**Case 2:** a healthy 24 year-old male was referred to our department for the extraction of decayed teeth. Radiographic investigation showed the projection of roots of 16, 17, 18 and 27 in the sinus (Figure 5). The 17 was decayed with presence of periapical radiolucency. After removal of the 17, oroantral communication was confirmed and its management was performed following the technique described in the first case report (Figure 6). Follow-up of the patient after six months showed a good healing without any complications (Figure 7).

## Discussion

The buccal advanced flap was performed to manage OAC/OAF in both case reports because of the simplicity of the technique and the presence of the suitable indication. The buccal sliding flap designed by Rehrmann [7, 8] is considered as the oldest and the most common surgical technique used for the treatment of OACs. This flap is developed by making two buccal divergent vertical incisions extending into the buccal vestibule from the extraction socket or from fistula orifice margins in case of OAF. The trapezoidal buccal flap is elevated and brought across the defect and sutured to the palatal margins of the defect [1]. In this procedure, a broad-based trapezoid mucoperiosteal flap is created and sutured over the defect. Its broad base assures adequate blood supply. Consequently, high success percentages (93%) have been reported [5, 9]. Disadvantages of the Rehrmann method includes the risk of reduction of the buccal sulcus depth and manifest postoperative pain and swelling. A prospective follow-up study by von Wowern [10] demonstrated that the reduction of sulcus depth after the Rehrmann method is permanent in half of the cases. An alternative method for closure of OACs is the Môczáir flap [11]; this method involves a buccal mucoperiosteal flap that is displaced one tooth width distally. The Môczáir flap is recommended for edentulous patients because the large denuded area, which is the result of the distal displacement of the buccal sliding flap, may give rise to periodontal disease in dentate patients. In addition, buccal sulcus depth is minimally influenced by advancement of the Môczáir flap in comparison with the Rehrmann method which may require an additional vestibuloplasty in denture wearing patients [5, 10]. In addition to the use of various flaps for closure, the use of some alloplastic materials has also been documented. Zide and Karas [12] used blocks of hydroxyapatite to close the communication by filling the bone defect in the alveoli. Due to patient's economic status, the option of using autogenous bone graft to fill the defect was kept for the 2nd surgery (if required); in case of failure of the flap alone to close the defect satisfactorily [1].

Since a chronic oroantral fistula can represent an access route for fungal infection, a systemic antifungal treatment must be used associated with abundant washings with saline and topical antifungal solution. Subsequently, a successful healing process requires absence of sinus infection and the advice of a specialist will help to deal with complications. If any are present, they must be treated with adequate nasal drainage. This kind of therapy might require a Caldwell-Luc procedure with nasal antrostomy or endoscopic sinus surgery [13]. Given the limitation of this technique of local flap for closure OAC, distant flaps and bone grafts can be used with success in the closure of large defects or in cases where local flaps have failed [14]. The use of biological material, alloplastic, or immediate implantation for the closure of OAC is usually indicated in the closer of OAC with a diameter of 3-4 mm provided that the maxillary sinus is uninfected or no foreign body is within the antrum [15]. Application of various synthetic materials like Bio-Oss-Bio-- Gide Sandwich technique has yielded excellent results for OAC closure. The technique achieves both bony and soft tissue closure, by contrast with only soft tissue closure obtained by local flaps [14, 16]. The presence of a large defect in the underlying bone that supports flap may cause the failure of closure of large ORFs [16]. Many techniques are used to reconstruct this bony defect, including metals, autogenous bone grafts, and nonporous hydroxyapatite blocks. Postoperative considerations consist on maintaining oral care, a diet of soft foods, the use of analgesics (e.g., non-steroidal anti-inflammatory drugs (NSAIDS)) and nasal decongestants which are recommended postoperatively. Further, nose blowing, sneezing with a closed mouth, and vigorous sports should be avoided [3, 14]. However, the two patients were fully satisfied and there were no recurrence symptoms of OAC/OAF present on 6th month's follow-up.

## Conclusion

Repairing oroantral defects is one of the most challenging and difficult problems in the field of oral and maxillofacial surgery. Multiple techniques are available from purely soft tissue flaps, which have proved to be successful over time, to a combination of hard tissue grafts (autologous, alloplastic, or allograft), which can be useful with the increased demand for implant restorations. The use of buccal advancement flap technique is suitable for closure of small and mild fistulas, and it remains the simplest procedure with less postoperative follow-up and good outcome.

## Competing interests

The authors declare no competing interests.
